# Collaborative chronic care model implementation within outpatient behavioral health care teams: qualitative results from a multisite trial using implementation facilitation

**DOI:** 10.1186/s43058-021-00133-w

**Published:** 2021-03-24

**Authors:** Jennifer L. Sullivan, Bo Kim, Christopher J. Miller, A. Rani Elwy, Karen L. Drummond, Samantha L. Connolly, Rachel P. Riendeau, Mark S. Bauer

**Affiliations:** 1 Center for Healthcare Organization and Implementation Research, VA Boston Healthcare System, 150 S. Huntington Ave (152M), Boston, MA USA; 2 Department of Health Law, Policy and Management, Boston University School of Public Health, 715 Albany St, Talbot Building, Boston, MA USA; 3 Department of Psychiatry, Harvard Medical School, Boston, MA USA; 4 Center for Healthcare Organization and Implementation Research, VA Bedford Healthcare System, 200 Springs Road (152), Bedford, MA USA; 5 Department of Psychiatry and Human Behavior, Warren Alpert Medical School, Brown University, Providence, RI USA; 6 Center for Mental Healthcare and Outcomes Research, Central Arkansas Veterans Healthcare System, North Little Rock, AR USA; 7 Department of Psychiatry, University of Arkansas for Medical Sciences College of Medicine, Little Rock, AR USA

**Keywords:** Chronic care, Mental health, Qualitative research

## Abstract

**Background:**

This paper reports on a qualitative evaluation of a hybrid type II stepped-wedge, cluster randomized trial using implementation facilitation to implement team-based care in the form of the collaborative chronic care model (CCM) in interdisciplinary outpatient mental health teams. The objective of this analysis is to compare the alignment of sites’ clinical processes with the CCM elements at baseline (time 1) and after 12 months of implementation facilitation (time 2) from the perspective of providers.

**Methods:**

We conducted semi-structured interviews to assess the extent to which six CCM elements were in place: work role redesign, patient self-management support, provider decision support, clinical information systems, linkages to community resources, and organizational/leadership support. Interviews were transcribed and a priori CCM elements were coded using a directed content analysis approach at times 1 and 2. We sought consensus on, and compared, the extent to which each CCM element was in place at times 1 and 2.

**Results:**

We conducted 27 and 31 telephone interviews at times 1 and 2, respectively, with outpatient mental health providers at nine participating sites. At time 1 and time 2, three CCM elements were most frequently present across the sites: work role redesign, patient self-management support, and provider decision support. The CCM elements with increased implementation from time 1 to time 2 were work role redesign, patient self-management support, and clinical information systems. For two CCM elements, linkages to community resources and organizational/leadership support, some sites had increased implementation at time 2 compared to time 1, while others had reductions. For the provider decision support element, we saw little change in the extent of its implementation.

**Conclusions:**

Sites increased the extent of implementation on several CCM elements. The most progress was made in the CCM elements where sites had CCM-aligned processes in place at time 1. Teams made progress on elements they could more easily control, such as work role redesign. Our results suggest that maximizing the benefits of CCM-based outpatient mental health care may require targeting resources and training toward specific CCM elements—especially in the use of clinical information systems and linking with community resources.

**Trial registration:**

Clinical Trials NCT02543840.

**Supplementary Information:**

The online version contains supplementary material available at 10.1186/s43058-021-00133-w.

Contributions to the literature

- This study represents the first qualitative description of how care processes aligned with the evidence-based CCM elements changed over time during an implementation trial in outpatient mental health care teams.
- These findings indicate the importance of assessing (1) structures and processes before an implementation intervention is put into place and (2) how sites make more progress in areas somewhat established prior to implementation.
- These data indicate that implementation facilitation can result in positive changes in the CCM elements.

## Background

In 2012, mental health leaders within the Department of Veterans Affairs (VA) launched the Behavioral Health Interdisciplinary Program (BHIP) to build effective interdisciplinary general outpatient mental health teams. These teams serve a panel of about a thousand Veteran patients and include between 5 and 10 team members (e.g., licensed mental health practitioners and non-licensed clinical/administrative support staff.) [1]. The guiding model for this initiative is the collaborative chronic care model (CCM), an evidence-based approach to structuring clinical care [2, 3] with the goal of ensuring coordinated, patient-centered, and anticipatory care. There are 6 elements in the CCM including work role redesign, patient self-management support, provider decision support, clinical information systems, linkages to community resources, and organization and leadership support [4]. The CCM originated in the 1990s as the Chronic Care Model for chronic medical illnesses treated in primary care [2, 3]. More recent work, however, has involved applying the principles of CCM-based care to mental health conditions in both primary care and specialty mental health settings. Recent reviews of over 50 controlled trials of CCM-based care have demonstrated that it can improve mental health outcomes and satisfaction with no added net costs [5, 6].

Despite this evidence base, implementing CCM-based care in real-world clinical settings has proven difficult. For example, Smith and colleagues [7] have identified barriers to CCM implementation including competing system-level priorities and the need for modified clinical workflows and tracking. Unützer et al. [8] found patient factors (e.g., depression severity), clinical factors (e.g., years as a CCM practices), and the amount of implementation support were associated with patient outcomes. These challenges may be especially relevant to the CCM elements of work role redesign, clinical information systems, and organization and leadership support. To our knowledge, however, no previous research has specifically set out to track the extent to which each of the CCM elements was pursued over the course of an implementation trial.

Between 2016 and 2018, as part of a partnered evaluation with the VA’s Office of Mental Health and Suicide Prevention (OMHSP), our team conducted a hybrid type II stepped wedge cluster randomized trial to implement the evidence-based CCM in BHIP teams. Given the positive evidence regarding the success of the CCM to improve health outcomes and satisfaction, VA adopted the CCM as the model for the project using a workbook-guided approach [9]. We utilized an evidence-based implementation strategy known as blended or external-internal facilitation, in which an external facilitator brings both content and process improvement expertise to a site and works closely with an internal facilitator, who offers the knowledge of the site’s organizational culture and existing procedures [10]. We found that Veterans who received care from teams implementing the CCM had significantly lower mental health hospital admission rates compared with their peers at the same sites who were treated by teams who had not implemented CCM. Additionally, mental health status improved among Veterans who were treated by teams and had three or more mental health conditions. Clinicians within the teams experienced significant improvement in two aspects of team functioning (role clarity and focus on team priorities) [11]. Given the support from operational partners and results from this trial, the VA plans to implement CCM-aligned general mental health teams more widely throughout the healthcare system [9].

In addition to understanding the effectiveness of CCM implementation, we also assessed whether CCM-based care can be implemented under general clinical practice conditions in general mental health clinics (the implementation portion of the hybrid type II design). As part of this trial, we conducted provider interviews to understand how facilitated implementation of CCM elements was associated with improvements in collaborative care. Miller et al. [12] explored how aligned care practices were to the CCM elements at the trial baseline and found that there were both consistent and inconsistent processes in place within all 6 CCM elements. Staff supported the goals of CCM-based care although the CCM elements were not addressed holistically or in an integrated way [12]. The objective of this follow-up study is to compare the alignment of sites’ clinical processes with the CCM elements at baseline (time 1) and after 12 months of implementation facilitation (time 2), from the perspective of providers. This study provides critical information to guide future implementation of CCM-based care utilizing implementation facilitation. This study is particularly timely as healthcare organizations, including the VA, continue to adopt collaborative care and team-based care more generally.

## Methodology

This study was approved by the VA Boston Healthcare System Institutional Review Board. Nine teams from nine different Veterans Affairs Medical Centers (VAMCs) participated in this CCM implementation project; all participating teams received implementation facilitation support during the trial but the start time of that facilitation was staggered per the stepped-wedge design [11]. There were 3 facilitators and each facilitator was assigned to three sites. For each site, facilitation involved an in-depth pre-site visit assessment and orientation of the site to the facilitation process and the CCM; 1½-day face-to-face site visit by the external facilitator to launch the implementation; 6 months of weekly videoconferences and/or conference calls with the team, weekly meetings between the external and internal facilitators, and ad hoc telephone and email communications; and 6 months of step-down facilitation contacts as needed (sites varied in the degree to which they used step-down facilitation). Sites were assigned to study waves using a balancing algorithm [13]. Thus, sites that were waiting for implementation facilitation to begin served as controls; during their control condition phase, they received technical assistance, including the distribution of a BHIP-CCM Enhancement Guide (i.e., workbook based on CCM elements) (available upon request) as well as monthly conference calls. These calls were attended by the internal facilitator from each site and anyone else that the internal facilitator decided to extend the invitation to (most commonly active members of the site’s team). Attendance by those other than the internal facilitators was not regular. The initial few monthly calls were led by the external facilitators to introduce the CCM and implementation overview, followed by subsequent calls being discussion-oriented with sites sharing their experiences and asking questions of one another regarding how CCM can be best operationalized.

### Data collection

We conducted semi-structured qualitative interviews with team members at the nine VAMC sites at two time points—first at baseline before external facilitators actively engaged with the sites (time 1) and then after facilitation support had concluded (time 2). Time 1 interviews were scheduled as close to the start of each wave as possible—5 sites within the same month as facilitation and 4 sites within 1 month after the start of facilitation. The interviewers were members of the larger study team but separate from the implementation facilitation intervention and had no interaction with BHIP team members at the 9 sites outside of the data collection efforts. We recruited participants via email and sent up to 3 emails to BHIP staff participating in the CCM implementation project. The interview questions focused on the extent to which team processes and patient care were aligned with CCM-based principles including work role redesign, patient self-management support, provider decision support, clinical information systems, linkages to community resources, and organization and leadership support (see Table 1 for the codebook of CCM elements, definitions, and examples; see Supplemental file for interview guide). During time 2 interviews, we asked additional questions about whether any changes made during the project could be attributed to implementation facilitation (as opposed to other ongoing projects, initiatives, or contextual factors such as staff hiring or clinic reorganizations). Interviews lasted 30–60 min depending upon participant availability and were digitally audio-recorded and professionally transcribed verbatim. Two participants did not want to be recorded and notes were taken. Interviewers had background in health services research, implementation science, and qualitative methods.

**Table 1:** Codebook of collaborative chronic care model elements, definitions, and examples

CCM element	Definition
Organization and leadership support	Providing resources and involvement to the BHIP teams. It can come from various levels within the organization including executive level leaders as well as more direct line supervisors and managers in mental health specialty care services. *Example:* dedicating time to BHIP team meetings and incentivizing attendance; celebrating BHIP team successes; ensuring that BHIP teams are fully staffed and have access to the supports needed to enact the other CCM elements.
Work role redesign	Providing care that anticipates patients’ needs and preferences through redesign processes within an interdisciplinary team structure. *Example:* in many randomized trials, a care manager role is established to conduct phone-based assessments with patients, place reminder calls, and follow up after appointments to ensure continuity of care.
Patient self-management support	Enhancing Veterans’ self-management skills to help them work toward wellness outside of treatment sessions. *Example:* treatment contracts addressing self-management steps or coping skills for patients to use between appointments.
Provider decision support	Making sure the treatment team or the providers have access to needed clinical expertise. *Example:* provision of treatment manuals, medication algorithms, and streamlined access to specialty consultation (in cases where there is a concern outside of their particular area of expertise).
Clinical information systems	Using electronic/automated mechanisms to enhance evaluation and coordination of care, with an emphasis on caring for patient populations or panels. *Example:* a BHIP team may have an established registry or panel of patients for whom the team is responsible. Once a registry is established, the team can track outcomes across the whole team’s caseload to provide targeted feedback to providers.
Community linkages	Facilitated or systematic relationships outside of VA to support care delivery *Example:* routine use of local or web-based peer support services located outside of the clinical setting (e.g., Alcoholics Anonymous, National Alliance on Mental Illness)

### Data analysis

**Fig. 1:**
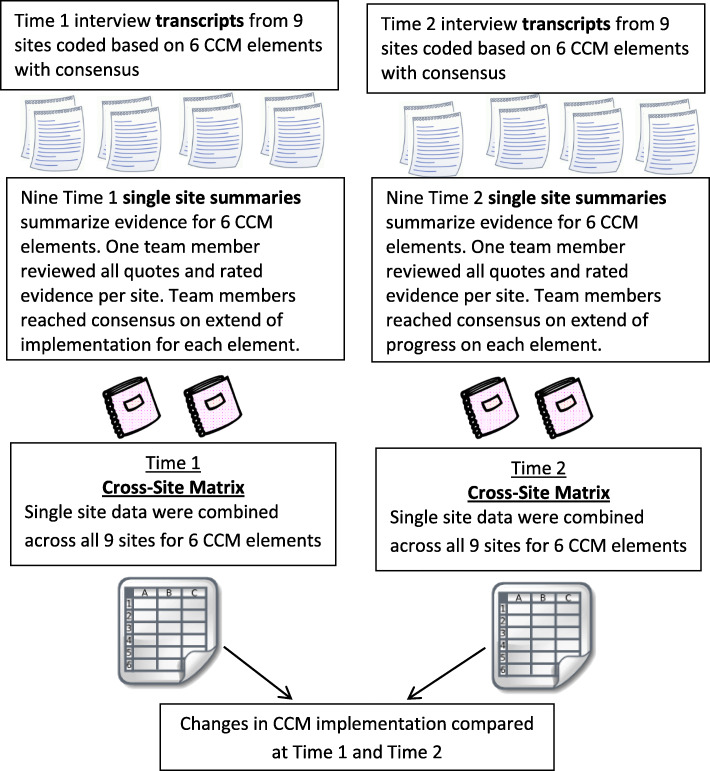
Work flow for data analysis: time 1 and time 2

Transcripts were coded using a directed content analysis approach [14] where we constructed an a priori framework of codes developed from the six CCM elements using NVIVO 10 software. Figure 1 displays our data analysis workflow. Initially, qualitative analysis team members coded the same three randomly selected transcripts independently. Our team met weekly over the course of early coding to establish a reliable and valid coding framework through discussion and consensus.

After determining the framework was reliable and valid, two team members independently coded each transcript using this framework and then met to come to consensus on any coding disagreements. Coding dyad assignments rotated among six team members (JS, BK, CM, KD, SC, ARE). Once coding was completed for each site, one team member created a descriptive summary of the evidence for each CCM element across all respondents at that site and included exemplar quotes for each element. Upon completion of summaries for each CCM element, the same coding team member categorized the extent to which each CCM element had been implemented. The categories we used included “little to no evidence present,” “inconsistently present,” “stably to narrowly present,” “several aspects stably present,” or “stably and broadly established.” Each completed site summary (including both text and ratings) was then reviewed by the larger team (JS, BK, CM, KD, SC, ARE) to come to consensus on the extent each CCM element had been implemented at each site. The ratings were discussed until our group of 5 team members participating in the meetings came to 100% consensus. After reaching consensus on nine site summaries, the study team created a cross-site matrix to compare the extent of CCM implementation across sites [15].

## Results

We conducted 27 interviews at time 1 and 31 interviews at time 2. Of the 58 interviews completed, 39 of these were conducted with unique respondents (i.e., only interviewed at time 1 or time 2). Thus, 19 participants were interviewed at both time 1 and time 2. Each site had between one and five respondents per timepoint. In total, 91 BHIP team members at the 9 sites were invited to participate. There were 33 team members who did not participate in the study—7 declined participation, 2 left the VA, and 24 did not respond after three recruitment emails. All 58 transcripts were included in the analysis for this manuscript. Our sample of 39 BHIP staff consisted of 11 social workers (28%), 9 psychologists (24%), 6 registered nurses (14%), 4 psychiatrists (10%), 4 vocational rehabilitation counselors (10%), and 5 other disciplines on the CCM implementation teams (e.g., addiction counselors, peer support specialists; 14%) participated in this study. Respondents had been involved in providing VA mental health services for a median of 10–15 years (ranging from 2 months to 25 years). In addition, respondents had been on the team for a median of 1.5 years (ranging from 2 months to about 2 years).

At time 1 and time 2, three CCM elements were most frequently present across the sites: work role redesign, patient self-management support, and provider decision support. The CCM elements with increased implementation from time 1 to time 2 were work role redesign, patient self-management support, and clinical information systems. Table 2 displays the extent of CCM element implementation at time 1 and time 2 across all sites. We observed that there was heterogeneity across sites across all CCM elements. Two CCM elements had a mixed change in the extent of implementation, meaning that there were some sites with increased implementation and others with reduced implementation in comparison to time 1. Specifically, within the community linkages element, three sites had an increase in the extent of implementation while two sites had a decrease in the extent of implementation. Similarly, for the organizational leadership and support element, one site had an increase in the extent of implementation while two sites had a decrease in the extent of implementation. The provider decision support element had few changes in the extent of implementation during our study period. In the following sections, we highlight the elements where there was increased or mixed change in the extent of implementation. Table 3 describes CCM element changes participants attributed to the implementation facilitation and Table 4 includes sample quotes for each CCM element discussed below.

**Table 2:**
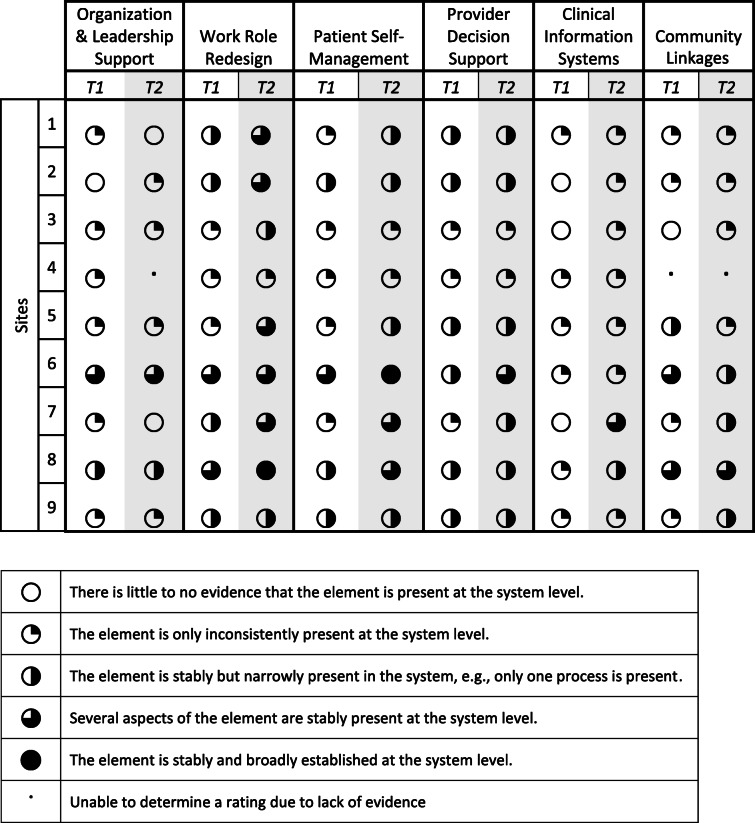
Extent of collaborative chronic care model implementation at time 1 and time 2

**Table 3:** Description of collaborative chronic care model element implementation attributed to the BHIP Enhancement Project

CCM element	Time 1	Time 2 changes
**Increase in extent of implementation**		
Work role redesign	No show procedures Same day access BHIP team meetings A great deal of informal communication	BHIP meetings increase BHIP team composition changes Discussions about improving team functioning, cohesion, coordination Improved communication
Patient self-management	Evidence-based practices Telephone contact with patients Completing treatment plans with patients	Patients attending BHIP team meetings Creation of additional educational materials
Clinical information systems	Use of Clinical Reminders Minimal use of patient panels	More discussion about having patient panels More discussion about measurement-based care
**Mixed extent of implementation (some sites increase, some sites decrease)**		
Community linkages	BHIP team members coordinate resources individually and also consult with staff with more knowledge of available resources	More efforts to coordinate across the BHIP team and have shared information on resources including development and use of team community resource lists
Organization and leadership support	Leaders broadly support project through resources and messaging Some leaders less directive and involved	Some supportive leaders More discussion of lack of leadership follow-through with resources A few staff mention active non-support for BHIP
**Little to no change in extent of implementation**		
Provider decision support	Use of evidence-based psychotherapies to treat patients Use of procedures to consult with providers with knowledge outside team members expertise Use of referral procedures to specialty care	Evidence-based trainings still occurring Limited evidence of some improvements in communication with some providers Limited evidence of increased knowledge of provider expertise within the team

**Table 4:** Exemplar quotes by collaborative chronic care model element and extent of implementation

CCM element	Sample quotes
**Increase in extent of implementation**	
Work role redesign	● “We have very experienced competent people on our team who know how [team-based care] should be delivered. This was a golden opportunity to start doing some of the things we accumulatively knew needed to happen” (Psychologist Time 2)
	● “What this project did was address longstanding problems and issues that we’ve had in the clinic that have gone unaddressed…the CCM forced us to address some issues … like how to make referrals, what our discharge criteria are, when to refer to specialty programs… just a lot of very subjective things that we have made more objective and made processes more efficient” (Psychologist Time 2)
Patient self-management	● “We actually have the patient join our team meetings which we’ve never done here before. We sat down with the patient and discussed [treatment] options so that the patient knew everybody on the team and so we are all on the same page” (Nurse Time 2)
	● “Yes, the BHIP team provides evidenced-based treatments and there are some additional evidenced-based therapies that are being introduced” (Psychiatrist Time 2)
Clinical information systems	● “I pay more attention to the panel management tool where I can look at the patient inactivity report…and I can look to see whether patients have been seen in the last two years and then we can look at potentially discharging them due to inactivity…whereas before I didn’t have access to this type of data” (Psychologist Time 2)
	● “We’ve starting to think about measurement-based care here…it’s been a topic of conversation within our team… I know we are exploring various measurements that we could use or we think would be beneficial” (Vocational Rehabilitation Counselor Time 2)
**Mixed change in extent of implementation**	
Community linkages	● “We’re doing a better job of informing our patients about community resources. We actually have a community resource guide that we’re able to share with patients now…it’s available for all providers on the team” (Psychologist Time 2)
	● “The social worker that left was one that was very versed in pretty much everything…so when we needed a community resource and we didn’t know, we would go to her. That person is gone and even though we have her contact list, you can’t figure it all out. There are probably things we’re not aware of.” (Nurse Time 2)
Organization and leadership support	● “I spoke with one executive leader and she complimented our work, so I know they are aware but they are not right up in our business” (Nurse Time 2)
	● “Executive leadership really doesn’t get it…they kind of miss the mark and they are looking at numbers versus people…facts versus what’s helpful and healthy for the staff” (Psychologist Time 2)
	● “One of the things we learned is getting your leadership on board is pretty important with this implementation because you need them involved in some of the CCM components like liaising across services… unfortunately the leader supporting us left and it’s gone in a different direction…leadership is shooting down certain aspects of [the CCM] which is a bit disempowering” (Psychologist Time 2)
**Little change in the extent of implementation**	
Provider decision support	● “our team has been extremely well trained…our therapists, each of them, have at least one of the VA evidence-based therapies under their belt. Most of them have more than one. So we have CBTI, a specialist and somebody that's CBT pain and then we have interpersonal therapy. So anyway, I don't think there's any new ones but we were really well stacked in that to begin with.” (Vocational Rehabilitation Specialist Time 2)
	● “not really any changes in expert consultation except that a lot of times in the past before [CCM implementation], like whoever met with resistance from another consulting service that wouldn’t take our patient or something, it was just kind of – it would just stop and now if there’s some resistance, one of us might bring it to the team and then as a team we’ll – the team decides ‘no, it’s still a good idea’, then we’ll document that and it’s been able to help us get more care for the patient than not just one person recommending person, it’s all of us….. I feel more comfortable reaching out to other disciplines because of the teams we have the backup there… I’m closer to my own team knowing what resources they can provide”. (Clinical Pharmacist Specialist Time 2)

### CCM elements with increased extent of implementation

#### Work role redesign

Participants felt that CCM implementation provided them with the time to focus on redesigning care processes that allowed teams to become more efficient. At time 1, staff mentioned having no-show procedures and same-day access procedures in place. Furthermore, time 1 interviews indicated that most teams had regular meeting times established although attendance was variable, and a significant portion of within-team communication occurred informally outside of team meetings. At time 2, staff still discussed same-day patient access and no-show procedures; however, there was more discussion around improved team functioning, cohesion, and coordination. Many respondents discussed developing team structures such as formalized intake procedures for patients, brief huddles for staff, and deliberate changes in team composition or work roles to reduce care fragmentation.

#### Patient self-management support

Time 1 interviews revealed an emphasis on evidence-based practices involving self-management components (e.g., cognitive behavioral therapy), telephone contact with patients, and use of treatment plans. At time 2, in addition to themes presented above, staff from several sites noted that patients were now attending team meetings to discuss their care. Teams also conducted additional patient education, such as providing brochures or informational packets to patients on the team describing available services and team structure.

#### Clinical information systems

At time 1, many staff mentioned use of clinical reminders to ensure that required screening tools were used consistently. There was limited evidence of patients and their health information being reviewed collectively as a panel by the teams, and cases were instead considered more often on an individual basis. Additionally, registry-based care where the team can track outcomes across the whole team’s caseload to provide targeted feedback to providers was only highlighted by one provider. At time 2, staff expressed more interest in tracking patients using a patient registry even though most of the teams had not begun utilizing patient registries. At time 2, providers at over half of the sites noted ongoing or upcoming efforts to incorporate measurement-based care into routine practice, involving administration of clinical assessments to guide ongoing care.

### CCM elements with mixed change in extent of implementation

#### Community resources

At time 1, staff discussed having inconsistent knowledge about community resources such as Vet Centers (which offer outreach, counseling, and referral services to eligible veterans), Alcoholics Anonymous groups, and Veteran Service Organizations. Additionally, referral to community providers was most frequently described as an individual process where providers would make their own referrals based on their knowledge of resources or would get expert consultation from other staff members within or outside of their team. At time 2, familiarity with community resources still varied across team members and providers often were still handling referrals individually. However, at some sites, progress was made on creating and periodically updating guides of available community resources for the teams to use. We also found team meetings provided a structure for more communication about community resources as a team. There were two sites where the extent of implementation decreased due to knowledgeable staff leaving the job or difficulty building community partnerships.

#### Organizational and leadership support

At time 1, staff at many sites felt that leaders broadly supported the implementation of CCM-based care through resources (e.g., staffing and training) and through having the CCM implemented. Some sites described having leaders who were less directive and less involved with CCM implementation. Often times, team members recognized that different levels of leadership were involved in CCM implementation and that direct supervisors or middle managers created a linkage between executive leaders’ expectations and team member needs. However, at time 2, we found more mixed viewpoints about leadership support for CCM implementation. For example, although some staff reported very supportive leaders, there were more reports of leaders not following through with resources or, in one case, actively not supporting the project.

### CCM elements with little to no change in extent of implementation

#### Provider decision support

At time 1, some staff (e.g., psychologists and social workers) at all 9 sites mentioned utilizing evidence-based psychotherapies to provide care to their patients. In addition, medication algorithms were available at all sites and used to assist in making decisions regarding prescriptions. Sites were more variable in the procedures providers used to seek recommendations from clinical experts and for referring patients to specialty services when needed. At time 2, staff reported very similar levels of implementation within this CCM element. At two sites, there were some limited improvements in information sharing regarding providers’ knowledge and expertise within the team as well as participation from additional staff outside the team to better coordinate care. A few staff mentioned receiving new evidence-based psychotherapy trainings at time 2, but not enough of a change in training to precipitate a change in the extent of implementation at time 2.

## Discussion

In this paper, we have described qualitative analyses regarding the extent to which the six evidence-based elements of CCM-based care were implemented in nine outpatient general mental health clinics. This work builds on our prior quantitative [11] and qualitative analyses [12]. To our knowledge, our study is the first to use directed content analysis [14] to investigate change over time in CCM implementation.

After 12 months of implementation facilitation [10], we found evidence for increased implementation of several CCM elements. This finding appeared strongest for the CCM elements for which infrastructure was already in place at time 1; this likely supported the element’s eventual improved implementation by time 2. For instance, for the element of work role redesign, the start of regular CCM team meetings at time 1 helped bring more structure to previously informal communication among team members, evidenced by improved team communication seen at time 2. For the element of patient self-management, emphasis on evidence-based practices that focus on patient self-management, as well as communicating with patients flexibly (e.g., via telephone) and collaboratively (e.g., completing treatment plans together), was already under way at time 1. At time 2, the evidence of patients actually attending CCM team meetings can be seen as an extension of the pre-existing collaborative approach to involving patients in their own care. For the element of clinical information systems, clinical reminders already existed at time 1 to help ensure that required screenings and symptom measurements are conducted regularly. Then at time 2, we saw of evidence of increased discussion of measurement-based care, which is about actually using the conducted measurements to actively shape care delivery.

We saw little change in the extent of implementation after 12 months of implementation facilitation for the provider decision support CCM element. Although staff consistently utilized evidence-based psychotherapies, medication algorithms, and standardized referrals, there were limited gains in integrated information sharing mechanisms both within the team and outside the team. However, teams felt they had established techniques for obtaining the expertise they needed. At time 1, we found that spontaneous discussions and outreach were the modalities for information exchange which is consistent with the concepts of healthcare teamwork [16]. These results suggest the importance of strong interpersonal relationships among team members and other interfacing providers in line with relational coordination [17]. Further, the use of personal coordination was supplemented with more formal standardized coordination (e.g., referrals placed through the electronic health record) suggesting the importance of both types of coordination in communicating within the team and with other providers outside of the team [18].

Our results indicate that when an implementation effort was perceived to have some leadership support at the start of implementation, it did not necessarily mean that the perception held at the end of implementation. Demonstrated leadership support (e.g., engagement, involvement, and actions) has been shown to be a distinguishing factor in sustainability [19, 20]. Thus, organizations that are looking to sustainably implement the CCM may want to plan and conduct explicit and ongoing steps to collaborate with leadership to help support front-line implementation teams throughout the entire duration of implementation. Although team members can take steps to try and convince leadership through meetings, presentations, and data on patient satisfaction or clinical outcomes, leadership ultimately make their decision about support based on other contextual factors outside of the general mental health team. Thus, teams need to think carefully about how best to engage leadership to maximize leadership support especially focused on middle managers who often make decisions about resources and training in line with executive leadership’s vision. Limited funding and resources have been found to be barriers to sustainment in previous studies [21], and the implementation science field is increasingly calling for an increased focus on mechanisms that specifically promote sustainability [22]. To this end, we are currently pursuing recently funded subsequent work trialing CCM implementation that targets sustainment of CCM elements beyond the duration of active facilitation.

Our study had several strengths and limitations. This work was part of a rigorous implementation trial collecting data both on health outcomes of CCM implementation in general mental health clinics, as well as implementation outcomes such as CCM-based clinical impacts over a 1-year period. Thus, we have a rich dataset available in which to put the CCM implementation intervention in context. In addition, this study represents the first qualitative description of how care processes aligned with the evidence-based CCM elements changed over time during a trial in outpatient mental health care teams. However, there were also some limitations to report. The number of participating team members varied by site, as did the staff interviewed at both time points. In cases where sites had a small number of respondents, we were cautious in interpreting CCM elements’ extent of implementation. Also, we were not able to examine whether underlying CCM-oriented care practices were a result of the monthly conference calls that were a part of the control condition through monitoring of these conference calls and identifying practices that result from these calls that may have served as implementation strategies themselves. Future studies ought to consider explicitly incorporating such examination. Finally, we focused available study resources on assessing the extent of CCM implementation only from the perspective of BHIP-CCM team members. We might have gained additional insights on CCM implementation by including other stakeholder perceptions outside of the team.

There are several suggestions for future work to build upon current findings. Given renewed interest in identifying the mechanisms by which healthcare processes and implementation strategies exert their effects, studies that explicitly elucidate the relationship between CCM implementation and clinical outcomes are needed. For instance, building on our finding that perceived leadership support at the beginning of implementation did not necessarily hold at the end of implementation, subsequent research should assess the impact of change in leadership support on clinical outcomes over time. Additionally, further work should examine how care structures and processes based on the CCM can be incorporated into general mental health care delivery beyond the VA, especially given how contexts (e.g., available resources) may vary widely among healthcare systems. Also, whether the extent of the step-down facilitation effort (which varied across sites for this study) impacts sustainment of CCM elements is an open question; as mentioned above, we currently have a subsequent study under way that focuses on CCM sustainment, and we look forward to assessing this relationship as part of that work. Finally, more work needs to be done to assess the type, amount, and content of implementation facilitation support needed by contextually different sites implementing the CCM.

## Conclusions

While considering our findings’ implications for future CCM implementation, we revisited the implications that we previously reported [12], to carefully reflect on whether any updates are needed based on this current study’s findings. The first of three previously reported implications was that many respondents were in favor of the overarching goal of CCM-based care (i.e., care delivery that is more coordinated, anticipatory, and evidence-based). We found this to consistently be the case following 12 months of implementation, demonstrated through (i) increased team structures and processes, (ii) communication of the structures and available services to Veterans, and (iii) incorporation of routine clinical assessments to guide ongoing care. These changes also speak to the second previously reported implication, of CCM elements being pursued separately from formal systems and structures. Namely, they exemplify the formalization of previously more impromptu discussions and outreach that characterized the work of our participating provider teams. The third of the three previously reported implications was the need for adequate human resources, funding, and workforce development for CCM implementation. Findings from this current study reiterate this need, as they highlight participants’ perspectives on (i) knowledgeable staff being key to maintaining community partnerships, (ii) whether their leadership has delivered on promised resources, and (iii) experience with trainings for enhancing staff’s abilities to deliver evidence-based care. In this study, we observed that all CCM elements required participation from staff and stakeholders outside the team to make progress on CCM elements. In this study, the distinction between processes that were within versus outside of the team’s control was particularly important. Our results indicate the salience of stakeholder engagement, building collaborations across services, and identifying processes that may be difficult to tackle because they require many people with different priorities to work in concert.

## Supplementary Information

**Additional file 1.** Interview guide.

## Data Availability

The data analyzed during the current study are not publicly available because individual privacy could be compromised.

## Abbreviations

BHIP: Behavioral Health Interdisciplinary Program
CBT: Cognitive Behavioral Therapy
CCM: Collaborative Chronic Care model
EBP: Evidence-Based Practice
EHR: Electronic Health Record
OMHSP: Office of Mental Health and Suicide Prevention
VA: Department of Veterans Affairs
VAMC: Veterans Affairs Medical Center
