# Supplementary material for: Collaborative chronic care model implementation within outpatient behavioral health care teams: qualitative results from a multisite trial using implementation facilitation
Source: Implement Sci Commun. 2021 Mar 24;2:33. doi: 10.1186/s43058-021-00133-w (PMC7992349; doi:10.1186/s43058-021-00133-w)
Supplement: Supplementary file 1 — Additional file 1. Interview guide. [file 43058_2021_133_MOESM1_ESM.docx]

**Supplemental Materials: Time 2 Interview Guide**

*At Time 1, the same questions were asked without the phrase “:Compared to 12 months ago” In Time 2, we also added the General Impressions questions as well as impressions about facilitation.*

| **Domain/Question** | **Construct/CCM Element** |
| --- | --- |
| **Part I: Background** | |
| 1. Before we start talking about care for veterans with mental health conditions, I’d like to briefly gather a little background about you. Could you tell me a little about your role here in the General Mental Health Clinic? | Background |
| **Part II: General Impressions (Time 2 only)**  Over the past 12 months since working on BHIP implementation, what changes if any has your team made?  Do you feel these changes are due to participation in this project or something else?  Are there any lessons your team has learned? If so, what are they? | |
| **Part III: General Mental Health Systems** | |
| 1. Compared to 12 months ago, when you are working with a complicated patient whose treatment requires input from multiple clinicians, how is care managed across all the providers who treat this patient? | Workrole redesign, guiding question |
| 1. Compared to 12 months ago, does your clinic currently deliver services in a team-based format?   *If so:*   - What disciplines are represented on the team? - How do you identify a shared caseload or panel that the team is jointly responsible for? - How are patients shared? - What services are available to the team’s patients? - How are patients educated about how the team functions?   - About what services are available to them through the team?   - About how to access which team members and when? - How much time is spent in team meetings (or huddles) to assure that patient care is well-coordinated? - Are there aspects of the physical space (or other resources) plant factors that enhance or impede working as a team (e.g., contiguous space or shared electronic resources)? | Workrole redesign |
| 1. Compared to 12 months ago, what types of things does your clinic do to ensure timely access to care for new or incoming patients?   *Follow-up questions can explore:*   - Availability of same-day appointments, team-based walk-in clinic times, or team-based “open-access” scheduling | Workrole redesign |
| 1. Compared to 12 months ago, what kind of support do you receive in staying informed about patient progress between appointments?   *Follow-up questions can explore:*   - Collaboration, communication and coverage among team clinicians - The role of information technology (e.g., regular reports from your clinic, secure messaging from staff or patients, MyHealtheVet) - Whether specific staff are tasked with follow-up for patients assigned to multiple providers - What mechanisms are in place to provide care between appointments for patients who may need follow-up to prevent worsening symptoms | Workrole redesign, Clinical information systems |
| 1. Compared to 12 months ago, tell me about how patients themselves may play an active role in the treatment process.   *Follow-up questions can explore:*   - How patient preferences and goals are incorporated into treatment planning - How patients learn about their condition(s) - How patients learn to manage their symptoms - How patients can provide feedback on the care they are receiving | Patient self-management support |
| 1. Compared to 12 months ago, what kinds of evidence or evidence-based treatments do you use when caring for your patients?   *Examples:* clinical practice guidelines, medication algorithms or evidence-based treatment manuals   - How do you get access to these resources? - Is there anything that your leadership, clinic, or hospital does to make it easier to obtain or use these resources? | Provider decision support  Clinical information systems |
| 1. Compared to 12 months ago, do you use measurement-based scales in the care of your patients?  - What types of measurements do you use? - *Examples:* - Depression, PTSD, Psychosis, Qualify of life scales? | Provider decision support  Clinical Information systems |

| 1. Compared to 12 months ago, what liaison mechanisms are in place when patients’ mental health care is provided in multiple settings or when care is being transitioned?     *Examples:*   - From your team to another specialty such as substance abuse or PTSD? - Between outpatient and inpatient care? - Discharge planning? | Workrole redesign |
| --- | --- |
| 1. Compared to 12 months ago, what do you do when you need access to expert consultation outside of mental health, i.e., referral to primary or specialty care, for an aspect of care beyond your area of expertise?   *If the respondent endorses availability of such consultation or referral processes:*   - How do you incorporate feedback or input from other providers into treatment planning? - Can you give some examples of resources inside and outside of your specific team that you use regularly? - Is there anything that your leadership, clinic, or hospital does to make it easier to seek this type of consultation or referral? | Provider decision support  Clinical information systems |
| 1. Compared to 12 months ago, how does your clinic link to and interact with resources outside of this hospital, for example the legal system, homeless shelters, police, Vet Centers, or groups like Alcoholics Anonymous?   *Follow-up questions:*   - How do you learn about available community resources? - Is contact information for community resources used by your service routinely compiled and available to clinicians? - How systematic are interactions with community resources? | Linkages to community resources |
| 1. Compared to 12 months ago, does your clinic have any way to track information across different patients? (Note that CPRS and Mental Health Assistant do not track information across patients.)   *Specific examples might include:*   - Routine collection of self-report measures to guide treatment - A registry or database that can inform you of patients seen in your clinic who may be experiencing symptoms or not responding as expected to treatment | Clinical information systems |

| 1. Compared to 12 months ago, do you receive feedback on the quality of care you provide to your patients, above and beyond annual performance reviews?   *If so, can inquire about:*   - Audit-based feedback regarding quality, timeliness, or personal performance on clinical reminder requirements - Timely and useful performance evaluations from peers or supervisors (again, above and beyond annual peer reviews) | Provider decision support  Clinical information systems |
| --- | --- |
| 1. Compared to 12 months ago, to what extent has BHIP implementation influenced the way you care for your patients?  - What factors are affecting how this change is going? - Is there someone in your group who is helping pull BHIP together? | Workrole redesign |
| 1. Compared to 12 months ago, can you tell me a bit more about how leadership supports (or hinders) your efforts to improve the quality, timeliness, and comprehensiveness of the care you deliver?   *Specific probes:*   - How does leadership support or hinder clinician efforts to pursue Quality Improvement trainings or projects? - How does leadership provide guidance to help you prioritize competing demands on your time? - Does leadership provide any financial incentives related to your performance? If so, how? | Leadership/ organization support |
| 1. Did your team work with an internal facilitator? Who was it? How did working with that person go? What went well? Were there things that could have been improved?   How did the process of coming together with the internal facilitator and the team go?  What did you like best about that time?  Do you have any suggestions for additional things you would have liked to do during that time? | Facilitation |
| 1. Did your team work with an external facilitator? Who was it? How did working with that person go? What went well? Were there things that could have been improved? | Facilitation |
| 1. If you could change anything about the way care is provided here, or include anything else about what this clinic might have or look like, what would that be? | Open-ended framing question |
